# Characteristics, treatment patterns, and biomarker testing of patients with advanced *RET* fusion-positive non-small cell lung cancer in a real-world multi-country observational study: a brief report

**DOI:** 10.3389/fonc.2025.1470387

**Published:** 2025-02-06

**Authors:** Urpo Kiiskinen, Grace Segall, Hollie Bailey, Cameron Forshaw, Tarun Puri

**Affiliations:** ^1^ Eli Lilly and Company, Indianapolis, IN, United States; ^2^ Adelphi Real World, Bollington, Cheshire, United Kingdom

**Keywords:** clinical characteristics, treatment patterns, biomarker testing, *RET* fusion-positive non-small cell lung cancer, real-world data

## Abstract

**Introduction:**

Approximately 1−2% of non-small cell lung cancers (NSCLCs) are positive for rearranged during transfection (*RET*) gene fusions. The aim of this real-world multi-national study was to describe clinical characteristics, biomarker testing, and treatment patterns of patients with *RET* fusion-positive NSCLC.

**Methods:**

This observational study was conducted in 2020 in nine countries using electronic patient record forms, following Adelphi Disease Specific Programme (DSP™) methodology. Patients with advanced NSCLC (aNSCLC) were included in the overall cohort. A smaller *RET* fusion-positive cohort comprised patients from the overall aNSCLC cohort who had *RET* fusion-positive disease and no other co-alterations, plus an oversample of patients with *RET* fusion-positive disease and no other co-alterations.

**Results:**

Patient characteristics were generally similar between the overall aNSCLC cohort (n=2947) and the *RET* fusion-positive cohort (n=576), aside from higher proportions of White/Caucasian patients, never smokers, and adenocarcinoma among the *RET* fusion-positive cohort. For the overall aNSCLC cohort, 899 (31%) were tested for *RET* fusions; 84% of *RET* test results were available prior to initiation of aNSCLC treatment. Comparisons between the two cohorts showed similar proportions of patients treated with chemotherapy (± immunotherapy), but less use of immunotherapy only or targeted therapy in the *RET* fusion-positive cohort.

**Conclusions:**

Results of this real-world study provide insights into clinical characteristics, biomarker testing, and treatment patterns of patients with *RET* fusion-positive aNSCLC and highlight the need for awareness and education to increase *RET* testing with the intent to treat with selective RET inhibitors when appropriate to optimize outcomes for patients.

## 1 Introduction

Non-small cell lung cancer (NSCLC) represents approximately 80−90% of all lung cancers (1, 2). Recently, treatment options for patients with advanced NSCLC (aNSCLC) have greatly expanded with the identification of targetable oncogenic driver alterations and the regulatory approval of several targeted therapies. One of these biomarkers is rearranged during transfection (*RET*) (3). Approximately 1−2% of all NSCLCs are positive for *RET* gene fusions (4, 5). Targeted treatments for *RET* fusion-positive aNSCLC include the selective RET kinase inhibitors selpercatinib and pralsetinib (6, 7).

Understanding real-world patient characteristics and treatment patterns, alongside more comprehensive information on biomarker testing, can provide important context for the rapidly evolving landscape of aNSCLC therapy and aid the generalizability of clinical trial data to routine clinical practice. The aim of this real-world multi-national study was to describe the clinical characteristics, biomarker testing, and treatment patterns of patients with *RET* fusion-positive aNSCLC.

## 2 Material and methods

This observational study was conducted from July to December 2020 in nine countries (Brazil, France, Germany, Italy, Japan, Spain, Taiwan, the UK, and the USA) following Adelphi DSP™ methodology, which involves large, multinational, cross-sectional surveys that collect real-world data from physicians and patients (8). Here, we report on patient-level data using electronic patient record forms (ePRFs) completed by physicians.

### 2.1 Recruitment, eligibility criteria, and data collection

Oncologists and pulmonologists (and respiratory surgeons in Japan) were identified using publicly available lists of clinicians in each country. Eligible specialists were responsible for managing patients with aNSCLC and saw at least three patients with a diagnosis of aNSCLC per month. A sample was then randomly selected from willing clinicians meeting the inclusion criteria.

**Figure 1:**
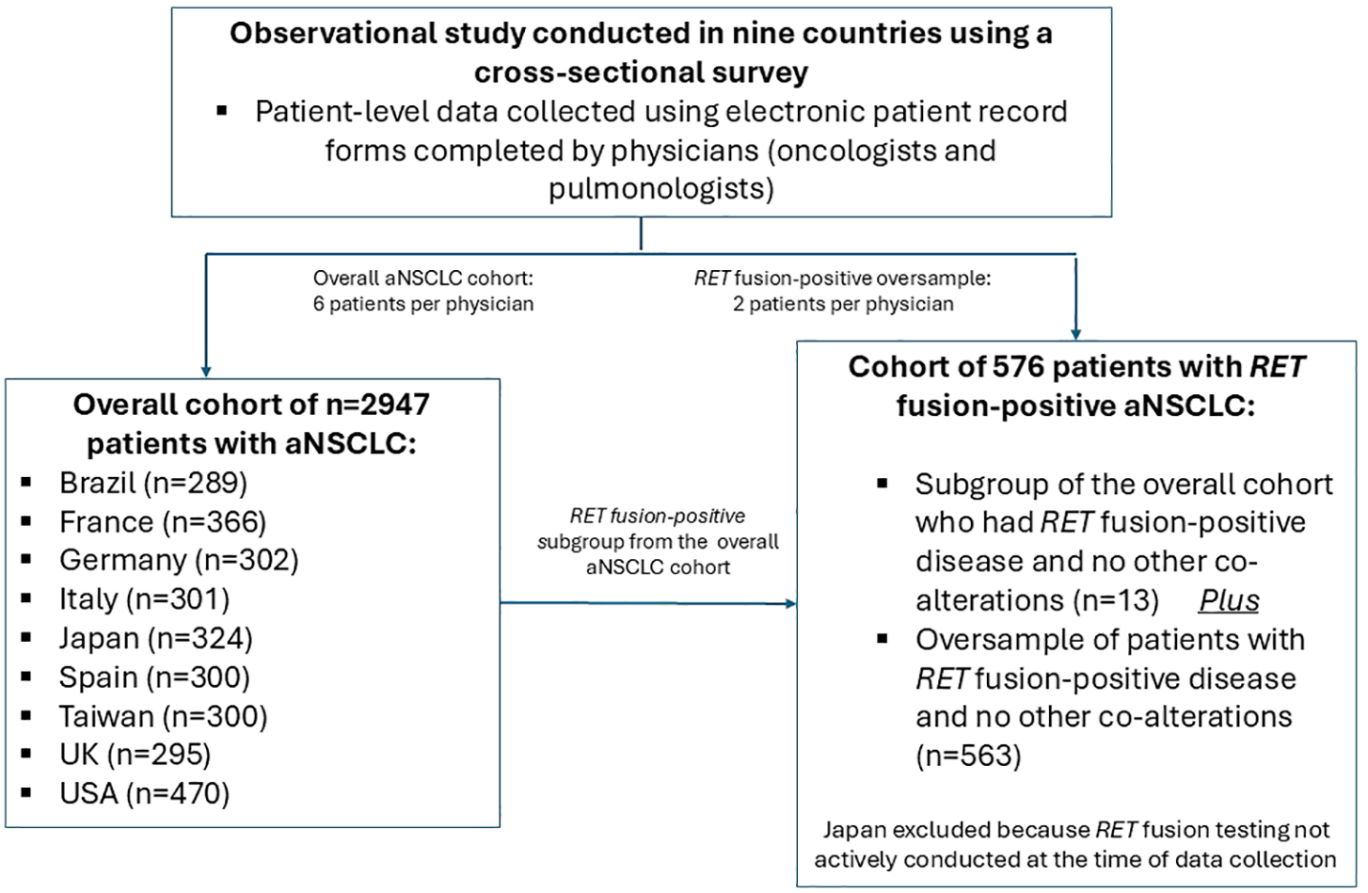
Schematic flow chart showing study design. aNSCLC, advanced non-small cell lung cancer; *RET*, rearranged during transfection.

Clinicians completed an online anonymized ePRF based on prior medical records for six consulting eligible patients with aNSCLC who were included in a pseudorandom sample (hereafter referred to as the overall aNSCLC cohort). An additional two patients were part of an oversample of patients with *RET* fusion-positive aNSCLC and no other co-alterations (hereafter referred to as the *RET* fusion-positive cohort). This latter cohort also included patients from the overall aNSCLC cohort whose disease was *RET* fusion-positive with no other co-alterations (**Figure 1**). Japanese patients were excluded from the *RET* fusion-positive cohort because *RET* fusion testing was not actively conducted in Japan at the time. Eligible patients were ≥18 years old, not participating in a clinical trial, and had a diagnosis of aNSCLC.

### 2.2 Study variables

Physicians provided information on patient demographics, clinical characteristics, biomarker testing, and first-line treatment. In particular, the ePRFs captured individual patient data on *RET* testing, including whether *RET* testing was conducted, results of *RET* testing, and whether next-generation sequencing (NGS) was used.

### 2.3 Statistical analyses

Descriptive statistics are provided for demographics and disease characteristics, using median with interquartile range (IQR) for continuous variables, and the frequency and percentage within each category for categorical variables. Missing data were excluded from the analysis and no imputation was conducted.

### 2.4 Ethical considerations

Data collection was in line with European Pharmaceutical Marketing Research Association guidelines (9). Study materials and protocol were reviewed and exempted by the Western Institutional Review Board (study protocol number AG8757) and were in full accordance with relevant legislation at the time of data collection (10, 11).

## 3 Results

Demographic and clinical characteristic data were available for 2947 patients in the overall aNSCLC cohort and 576 patients in the *RET* fusion-positive cohort (**Table 1**). Characteristics were generally similar between these cohorts for most parameters, including median age (67 [IQR 60−72] years vs. 65 [IQR 57−70] years), presence of central nervous system metastases, and programmed cell death ligand 1 (PD-L1) expression, and with most patients in each cohort being male, having stage IV disease and Eastern Cooperative Oncology Group performance status (ECOG PS) 0−1 (**Table 1**). Median age for the overall aNSCLC cohort was similar across the nine countries (data not shown).

**Table 1:** Patient demographic and clinical characteristics in the overall aNSCLC population and in patients with *RET* fusion-positive aNSCLC and no other co-alterations.

Characteristic	Overall aNSCLC (n=2947)^a^		*RET* fusion-positive aNSCLC^b^ (n=576)^a^	
Age, median (IQR), years	67	(60−72)	65	(57−70)
Sex, n (%)				
Male	1788	(61)	327	(57)
Female	1159	(39)	249	(43)
Ethnic origin, n (%)				
White/Caucasian	2017	(68)	486	(84)
Japanese	324	(11)	0	(0)
Han Chinese	293	(10)	27	(5)
African American	76	(3)	12	(2)
Hispanic/Latino	50	(2)	13	(2)
Other	187	(6)	38	(7)
Smoking status, n (%)				
Current smoker	455	(15)	46	(8)
Former smoker	1779	(60)	306	(53)
Never smoked	669	(23)	208	(36)
Unknown	44	(1)	16	(3)
Disease stage at initial diagnosis, n (%)				
Stage I	50	(2)	1	(<1)
Stage II	101	(3)	16	(3)
Stage III	634	(22)	81	(14)
IIIA	96	(3)	6	(1)
IIIB	337	(11)	33	(6)
IIIC	201	(7)	42	(7)
Stage IV	2138	(73)	471	(82)
Unknown/not assessed	24	(1)	7	(1)
Histology, n (%)				
Adenocarcinoma	2064	(70)	509	(88)
Squamous cell carcinoma	746	(25)	36	(6)
Large cell carcinoma	93	(3)	24	(4)
Other	33	(1)	6	(1)
Unknown/not assessed	11	(<1)	1	(<1)
ECOG PS score at advanced diagnosis, n (%)	(base n=2941)			
0−1	2292	(78)	458	(80)
2	431	(15)	69	(12)
≥3	183	(6)	44	(8)
Unknown/not assessed	35	(1)	5	(1)
Presence of CNS metastases at any time (primary site), n (%)	(base n=2439)		(base n=518)	
Brain	189	(8)	30	(6)
Other CNS	54	(2)	24	(5)
Presence of CNS metastases at any time (secondary site), n (%)	(base n=2439)		(base n=518)	
Brain	228	(9)	32	(6)
Other CNS	72	(3)	19	(4)
PD-L1 expression, n (%)				
<1%	560	(19)	179	(31)
1−49%	1182	(40)	248	(43)
≥50%	692	(23)	66	(11)
Unknown/not assessed	513	(17)	83	(14)

a Number of patients with data for each parameter unless indicated otherwise.

b Without other co-alterations.

aNSCLC, advanced non-small cell lung cancer; CNS, central nervous system; ECOG PS, European Cooperative Oncology Group performance status; IQR, interquartile range; PD-L1, programmed cell death ligand 1; *RET*, rearranged during transfection.

Although statistical comparisons were not made, there were notable numerical differences between the overall aNSCLC cohort and the *RET* fusion-positive cohort for some parameters, including higher proportions of White/Caucasian patients (68% vs. 84%), never smokers (23% vs. 36%), and histology of adenocarcinoma (70% vs. 88%), and lower proportion of squamous cell carcinoma (25% vs. 6%), in the *RET* fusion-positive cohort. All comparisons between cohorts came with a caveat that the *RET* fusion-positive cohort excluded Japanese patients and included a small number of patients from the overall cohort. In the *RET* fusion-positive cohort, there was wide variation in the proportion of never smokers across countries, ranging from 24% and 31% in France and the USA, respectively, to 70% in Taiwan (data not shown).

The testing rate for *RET* gene fusions at diagnosis of advanced disease was 31% (899 of 2947 patients) in the overall aNSCLC cohort, but varied widely across countries, from 8% in Taiwan to 67% in the USA (**Table 2**). Testing for epidermal growth factor receptor (*EGFR*) mutations was more consistent across countries, ranging from 73% in Brazil to 90% in Taiwan. In general, the USA had the highest rates of testing across all biomarkers (**Table 2**). For patients in the overall aNSCLC cohort with available results (n=820), the prevalence of *RET* gene fusions was 4.8%, but was as high as 26.8% in Brazil. Most patients (77%) had been tested using NGS, and 84% had test results available prior to initiation of treatment for advanced disease (**Table 2**).

**Table 2:** Biomarker testing for the overall aNSCLC cohort and breakdown by country.

Parameter	Overall aNSCLC (n=2947)^a^	USA (n=470)^a^	Brazil (n=289)^a^	UK (n=295)^a^	Italy (n=301)^a^	France (n=366)^a^	Spain (n=300)^a^	Germany (n=302)^a^	Taiwan (n=300)^a^	Japan (n=324)^a^
Biomarkers tested, n (%)										
RET	899 (31)	313 (67)	85 (29)	52 (18)	82 (27)	147 (40)	86 (29)	62 (21)	25 (8)	47 (15)
PD-L1	2487 (84)	392 (83)	216 (75)	277 (94)	278 (92)	349 (95)	288 (96)	218 (72)	220 (73)	249 (77)
EGFR	2461 (84)	419 (89)	210 (73)	235 (80)	266 (88)	297 (81)	251 (84)	246 (81)	270 (90)	267 (82)
ALK	2294 (78)	391 (83)	190 (66)	235 (80)	264 (88)	289 (79)	245 (82)	210 (70)	236 (79)	234 (72)
ROS1	1881 (64)	364 (77)	131 (45)	189 (64)	223 (74)	244 (67)	229 (76)	137 (45)	188 (63)	176 (54)
KRAS	1200 (41)	336 (71)	100 (35)	95 (32)	105 (35)	240 (66)	104 (35)	111 (37)	44 (15)	65 (20)
BRAF	1084 (37)	322 (69)	82 (28)	62 (21)	114 (38)	185 (51)	83 (28)	96 (32)	34 (11)	106 (33)
TRK	533 (18)	246 (52)	57 (20)	31 (11)	29 (10)	44 (12)	43 (14)	39 (13)	18 (6)	26 (8)
MET	784 (27)	284 (60)	75 (26)	40 (14)	64 (21)	136 (37)	58 (19)	45 (15)	27 (9)	55 (17)
HER2	689 (23)	266 (57)	71 (25)	31 (11)	46 (15)	114 (31)	45 (15)	48 (16)	22 (7)	46 (14)
NGS used, n^b^ (%)	(base n=820)	(base n=291)	(base n=71)	(base n=49)	(base n=75)	(base n=137)	(base n=74)	(base n=58)	(base n=19)	(base n=46)
Yes	628 (77)	251 (86)	58 (82)	27 (55)	51 (68)	99 (72)	41 (55)	51 (88)	13 (68)	37 (80)
No	192 (23)	40 (14)	13 (18)	22 (45)	24 (32)	38 (28)	33 (45)	7 (12)	6 (32)	9 (20)
Results of *RET* gene fusion assessment at advanced diagnosis, n (%)	(base n=820)	(base n=291)	(base n=71)	(base n=49)	(base n=75)	(base n=137)	(base n=74)	(base n=58)	(base n=19)	(base n=46)
Positive	39 (4.8)	8 (2.7)	19 (26.8)	3 (6.1)	1 (1.3)	3 (2.2)	0 (0)	2 (3.4)	1 (5.3)	2 (4.3)
Negative	781 (95.2)	283 (97.3)	52 (73.2)	46 (93.9)	74 (98.7)	134 (97.8)	74 (100)	56 (96.6)	18 (94.7)	44 (95.7)
*RET* test results available prior to initiation of therapy for advanced disease, n (%)	(base n=820)	(base n=291)	(base n=71)	(base n=49)	(base n=75)	(base n=137)	(base n=74)	(base n=58)	(base n=19)	(base n=46)
Yes	689 (84)	249 (86)	53 (75)	39 (80)	70 (93)	113 (82)	56 (76)	55 (95)	14 (74)	40 (87)
No	119 (15)	37 (13)	15 (21)	9 (18)	5 (7)	24 (18)	18 (24)	0 (0)	5 (26)	6 (13)
Unknown	12 (1)	5 (2)	3 (4)	1 (2)	0 (0)	0 (0)	0 (0)	3 (5)	0 (0)	0 (0)

a Number of patients with data for each parameter unless indicated otherwise.

b Base numbers reflect patients with aNSCLC who had RET-fusion test results (positive or negative) available at the time of data collection.

ALK, anaplastic lymphoma kinase; aNSCLC, advanced non-small cell lung cancer; BRAF, v-raf murine sarcoma viral oncogene homolog B1; EGFR, epidermal growth factor receptor; HER2, human epidermal growth factor receptor 2; KRAS, Kristen rat sarcoma; MET, mesenchymal epithelial transition factor receptor; NGS, next-generation sequencing; PD-L1, programmed cell death ligand 1; *RET*, rearranged during transfection; ROS1, c-ros oncogene 1; TRK, tropomysin receptor kinase.

The percentage of patients receiving chemotherapy (33% vs. 32%) or chemotherapy plus immunotherapy (19% vs. 18%) as first-line treatment was very similar between the overall aNSCLC cohort and the *RET* fusion-positive cohort (**Table 3**). In the *RET* fusion-positive cohort, across the eight countries evaluated, chemotherapy was prescribed for 9−57%, and chemotherapy plus immunotherapy for 7−33%, in the first-line setting (**Table 3**). Compared to the overall aNSCLC cohort, the percentage of patients treated with immunotherapy only (18% vs. 9%) or with targeted therapy (26% vs. 16%) was numerically lower in the *RET* fusion-positive cohort.

**Table 3:** First-line drug treatment class among the overall aNSCLC cohort and *RET* fusion-positive cohort and by country for the *RET* fusion-positive cohort.

Parameter	Overall aNSCLC cohort (n=2947)	*RET* fusion-positive cohort (n=576)	USA (n=106)	Brazil (n=70)	UK (n=88)	Italy (n=56)	France (n=67)	Spain (n=85)	Germany (n=77)	Taiwan (n=27)
Chemotherapy, n (%)	977 (33)	185 (32)	10 (9)	14 (20)	29 (33)	32 (57)	25 (37)	40 (47)	23 (30)	12 (44)
Chemotherapy plus immunotherapy, n (%)	548 (19)	104 (18)	13 (12)	9 (13)	29 (33)	6 (11)	13 (19)	17 (20)	15 (19)	2 (7)
Immunotherapy, n (%)	523 (18)	52 (9)	6 (6)	8 (11)	12 (14)	3 (5)	5 (7)	5 (6)	13 (17)	0 (0)
Targeted therapy (+/- other), n (%)	774 (26)	92 (16)	11 (10)	21 (30)	4 (5)	3 (5)	15 (22)	6 (7)	23 (30)	9 (33)
Best supportive care only, n (%)	51 (2)	14 (2)	1 (1)	7 (10)	2 (2)	1 (2)	1 (1)	0 (0)	1 (1)	1 (4)
Other, n (%)	70 (2)	129 (22)	65 (61)	11 (16)	12 (14)	11 (20)	8 (12)	17 (20)	2 (3)	3 (11)

aNSCLC, advanced non-small cell lung cancer; *RET*, rearranged during transfection.

## 4 Discussion

This observational study provides real-world data from nine countries on the clinical characteristics, biomarker testing, and treatment patterns of patients with aNSCLC, focusing on data from patients with *RET* fusion-positive aNSCLC and no other co-alterations. Demographic and clinical characteristics, such as median age (65 years; IQR 57−70), advanced disease stage at diagnosis, predominantly adenocarcinoma histology, and a relatively high proportion of never smokers among the *RET* fusion-positive cohort were generally as expected for this patient population (3, 6). However, other studies have reported a preponderance of female patients (12), whereas in our *RET* fusion-positive cohort, 43% were female.

The *RET* testing rate of 31% across the nine countries in this study is much lower than those for *EGFR* and *ALK* (≈80%), the more established biomarkers for NSCLC. This has various clinical implications. First, *RET* is an emerging biomarker (13); selective RET inhibitors have been available only for the last 4 years but at the time of writing are still not widely available or reimbursed across all lines of therapy among the countries in this study, including Brazil and Taiwan (no access across any line of therapy) and France and Italy (no access for first-line therapy). As access to selective RET inhibitors expands, we expect testing rates to increase. Second, education and awareness for *RET* as an actionable biomarker needs to be pursued, as evidence clearly suggests that patients derive benefit when tested for *RET* and treated, if appropriate, with a selective RET inhibitor in the first-line setting (14). Third, as more actionable biomarkers emerge for NSCLC, broad testing with NGS should become the norm and this will help increase testing rates across the spectrum for all biomarkers. The 4.8% positivity rate for *RET* fusions in the overall aNSCLC cohort who were tested was somewhat higher than the expected rate of ≈1−2% for *RET* fusion-positive aNSCLC (4, 5), although the positivity rate was reduced to 2.7% after excluding data from Brazil, which had a positivity rate much higher than any other country (26.8%). The exact reason for this difference is not known.

The proportion of patients treated with first-line chemotherapy or chemotherapy plus immunotherapy was almost the same in both cohorts. However, the use of targeted treatment was numerically lower in the *RET* fusion-positive cohort than in the overall aNSCLC cohort, possibly because selective RET inhibitors were just becoming available at the time of the study. The use of immunotherapy was also numerically lower in the *RET* fusion-positive cohort, which appears to be consistent with the lower rate of PD-L1 expression ≥50% in this cohort.

Limitations of this study include its observational design, making it subject to potential biases inherent in non-randomized research, such as selection bias and confounding factors that could influence treatment choices and outcomes. For example, selection of consecutive patients may have resulted in over-representation of patients who consult more frequently. In addition, there may have been potential biases in selecting the *RET* fusion-positive cohort. This cohort included both a subgroup of patients with *RET* fusion-positive disease and no other co-alterations from the overall aNSCLC cohort and an oversample of those with *RET* fusion-positive disease and no other co-alterations, which may not accurately reflect the broader population of *RET* fusion-positive aNSCLC patients with varying molecular profiles. The retrospective study design using electronic patient records may have provided incomplete or inconsistent data across different sites, potentially affecting the accuracy of clinical information. The study was conducted across nine countries, which may have led to variability in treatment patterns and access to biomarker testing due to differences in healthcare systems, guidelines, and resources. Although 31% of patients in the overall aNSCLC cohort were tested for *RET* fusions, this may not represent the full patient population, and the availability of *RET* testing could be limited in some regions or settings, leading to underreporting of *RET* fusion-positive cases. It is also noteworthy that the study mainly focused on descriptive analysis of clinical characteristics and treatment patterns, rather than directly evaluating the effectiveness of different treatments in the *RET* fusion-positive cohort. This study was conducted almost 4 years ago and, while we believe the findings are still relevant, a repeat study should be performed to assess the impact of the advancements in diagnostic and treatment paradigms (e.g., availability of selective RET inhibitors) over these years.

In conclusion, this study provides insights into the clinical characteristics, biomarker testing, and treatment patterns of patients with *RET* fusion-positive aNSCLC and highlights the need for increased *RET* testing rates, preferably with NGS, with the option to treat with selective RET inhibitors in the event of *RET* fusion-positive disease.

## Data Availability

The raw data supporting the conclusions of this article will be made available by the authors, without undue reservation.
